# *Heracleum sosnowskyi* seed development under the effect of exogenous application of GA_3_

**DOI:** 10.7717/peerj.6906

**Published:** 2019-05-09

**Authors:** Dalia Koryznienė, Sigita Jurkonienė, Tautvydas Žalnierius, Virgilija Gavelienė, Elžbieta Jankovska-Bortkevič, Nijolė Bareikienė, Vincas Būda

**Affiliations:** 1 Institute of Botany, Nature Research Centre, Vilnius, Lithuania; 2 Institute of Ecology, Nature Research Centre, Vilnius, Lithuania

**Keywords:** Auxin, Gibberellin, Invasive, Parthenocarpy, Seed germination, Sosnowsky’s hogweed

## Abstract

Numerous studies have demonstrated the impact of exogenous gibberellin on fleshy fruit formation, but the effect on dry fruits is not yet well known. To test the role of gibberellin (GA_3_) in dry fruit formation, we analysed the impact of exogenous GA_3_ on the invasive plant Sosnowsky’s hogweed (*H. sosnowskyi* Manden.) seed development and germination. Treatment of GA_3_ concentrations of 0.07 mM, 0.14 mM, 0.28 mM, 0.43 mM was applied to flowers at the early stage of development. Seeds were collected from treated satellite umbels. It was observed that GA_3_treatment did not have a significant effect on the size of *H. sosnowskyi* seeds, but caused various changes in their shape. The data on semi-thin longitudinal sections of *H. sosnowskyi* mericarps and SEM micrographs of embryos showed that the embryos in GA_3_ (0.43 mM) treated variants were at torpedo stage, while in control variants—mature embryos. The germination of seeds of each variant was estimated by burying them in the soil. Our studies indicated that GA_3_ application reduced the germination of *H. sosnowskyi* seed from 98.0% (control) to 16.5% (GA_3_ concentration 0.43 mM). It was assumed that exogenous application of GA_3_ had influence on the development of dry Sosnowsky’s hogweed seeds and could be used to inhibit the spread of this invasive plant.

## Introduction

Sosnowsky’s hogweed (*Heracleum sosnowskyi* Manden.) is an invasive alien herbaceous monocarpic, perennial, seed-propagated plant. Like other invasive alien plants, it is leading to a reduction in local plant biodiversity. In Europe, it has rapidly established in a variety of seminatural and man-made ecosystems, particularly in spaces along water basins, forest edges, roadsides, meadows, open forests and unmanaged urban areas (Gudžinskas & Rašomavičius, 2005; Kabuce & Priede, 2010; Baležentienė, Stankevičienė & Snieškienė, 2013). Additionally, it can cause considerable economic damage, sometimes also presenting a health hazard to humans. Contact with *H. sosnowskyi* as well as other invasive genetically close plant—*H. mantegazzianum*—can cause phytophotodermatitis, a serious skin inflammation caused by UV photo-activation of furanocoumarins present in the sap (Nielsen et al., 2005; Jakubowicz et al., 2012).

Different agro-technical and chemical measures are used to stop its spread, but there are no universal tools to stop these invasive plants, to reduce their impact or prevent future invasions (Erneberg, Dahl Jensen & Strandberg, 2003; Nielsen et al., 2007). Herbicides are quite effective method to control the spread of this plant, however, such treatment is not recommended in natural habitats (e.g., along riversides), because of a risk of toxicity to fish, algae and other water plants and animals. An unsprayed buffer zone of 5 m should be left adjacent to any river or other water body (Marcher, 2001).

It is necessary to look for environmentally friendly biological measures to control invasion of the plant. Sosnowsky’s hogweed is a monocarpic plant and can live for several years, although the mother plant dies after producing seed once. Studies on the soil seed-bank of *H. sosnowskyi* in Lithuania have demonstrated that seed density directly affects the density of seedlings in spring. It is known that *H. mantegazzianum* average seed-bank survival is very low only after five years (Moravcova et al., 2007b; Moravcova et al., 2018; Gudžinskas & Žalneravičius, 2018). In alien species, the ability to develop a persistent seed bank is associated with their ability to naturalize and become invasive. Thus, to stop the spread of Sosnowsky’s hogweed, it is appropriate to use the biologically active compounds to inhibit their seed formation, development and germination. Fruit parthenocarpy would be a solution to this problem.

Auxins and gibberellins are two classes of plant hormones that are mostly used to obtain parthenocarpy. Experimental data have shown that application of these plant growth regulators can trigger fruit set even without pollination and can induce parthenocarpic fruit development (Serrani et al., 2008; Dorcey et al., 2009; Ren et al., 2011; Aparna & Remko, 2012; Cheng et al., 2013; Jung et al., 2014; Mazzucato et al., 2015; Chen et al., 2017; Joldersma & Liu, 2018). For several fruit crops, parthenocarpy is very often obtained by breeding methods. The use of chemicals capable of inhibiting polar auxin transport has become a valuable and powerful tool in demonstrating the involvement of auxin transport in such processes. Two of the most frequently used inhibitors are 2,3,5-triiodobenzoic acid (TIBA) and phytotropin, 1-N-naphthylphthalamic acid (NPA) (Hamamoto et al., 1998; Serrani et al., 2010). According to the bulk of the experiments, GA and IAA-induced parthenocarpic fruit development has been observed mostly in fleshy fruit crops. However, there is still a significant lack of research into the effect of these hormones on parthenocarpy of many other crops.

To improve our understanding of the hormonal control to fruit set and seed development, we studied the influence of GA_3_ and auxin transport inhibitor TIBA on dry fruits of the *Apiaceae* family plant. Several studies have indicated that the exogenous application of plant growth regulators induces changes in morphology and histology of fruit during its development (Serrani et al., 2007a; Fuentes et al., 2012; Cheng et al., 2013).

The aim of this research was to inhibit *H. sosnowskyi* seed formation, development and germination or to obtain fruit parthenocarpy.

## Materials and Methods

### Plant material

Studies were carried out on fruits of Sosnowsky’s hogweed (*Heracleum sosnowskyi* Manden). As in other species of the *Apiaceae* family, these fruits are dry schizocarps that consist of two strongly flattened mericarps (seeds) (Jahodova et al., 2007b; Kabuce & Priede, 2010; Jakubowicz et al., 2012).

The investigated population of Sosnowsky’s hogweed was located near Vilnius (Lithuania) on formerly natural forest edge. The studied area occupied 0.415 ha and was located between 54°758587′N and 25°260138′E. Detailed description of the location is in Jurkonienė et al. (2016). For our research, satellite umbels were treated and mericarps were collected from randomly selected plants.

### Treatment

Application of bioactive chemical compound gibberellic acid (GA_3_) (SERVA) concentrations of 25 mg/l, 50 mg/l, 100 mg/l, 150 mg/l (0.07 mM, 0.14 mM, 0.28 mM, 0.43 mM) was applied to flowers at the early development stage in June, 2–4 days before flowering. In order to increase higher content of native GA, the terminal umbels of flowering plants were removed. To show auxin transport influence on the development of seeds, auxin transport inhibitor 2,3,5-triiodobenzoic acid (TIBA) (5*10^−5^ M) was used (Serrani et al., 2010). After treatment with TIBA, the phon of auxin was recovered by indolylacetic acid (IAA) (5*10^−5^ M) (8.75 mg/l). All the axillary buds that developed as a result of plant decapitation were removed to prevent competition with developing fruits (Serrani et al., 2010). All applications of bioactive chemical compounds were sprayed with hand sprayer to runoff. To exclude pollen-carrying insects, sprayed inflorescences were covered with bags for the period of pollination. Distilled water was used as a control.

**Figure 1:**
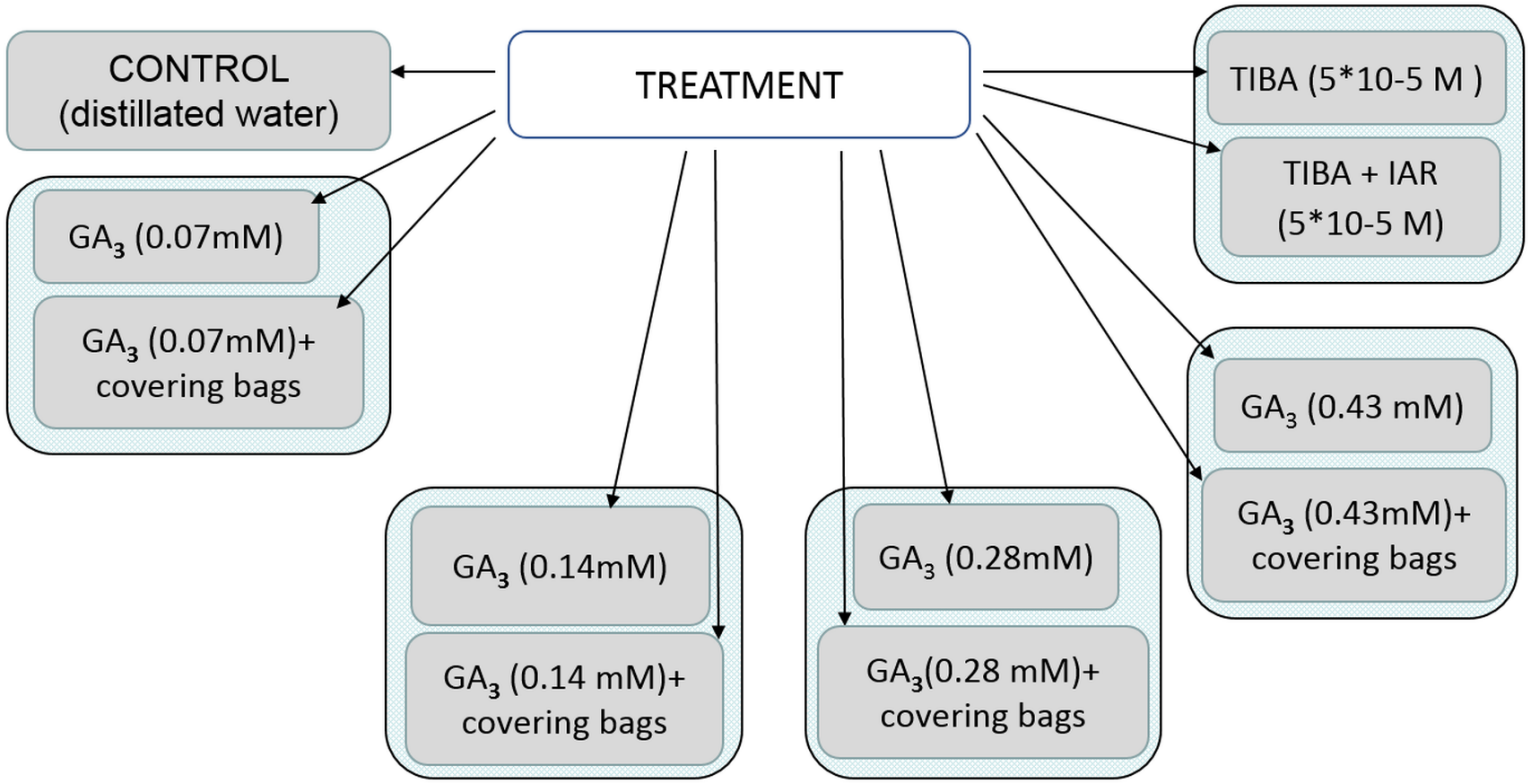
Scheme of treatment protocol. GA_3_, gibberellin; TIBA, auxin transport inhibitor 2,3,5,—triiodobenzoic acid; IAR, indolylacetic acid.

All the treatment protocol is shown in the scheme presented in Fig. 1.

A total of 9–10 plants and 25–30 inflorescences were used for flower treatment. Mature seeds were collected in late August in 2016–2017. The collected seeds were analysed immediately.

### Analysis of seed development

For anatomical evaluation, the seeds were composed in variac lines. Average samples were selected. A total of ten selected seeds of each variant were fixed in FAA solution (formalin-acetic acid-50% ethanol (1:1:20)) and left in a fridge for three days. Before transferring into a fresh portion of FAA, the fruit coat (pericarp) was removed. Then the samples were dehydrated in ethanol series and embedded in paraffin by standard procedures (Kublickienė, 1978). Longitudinal sections of seeds (5–7 µm thick) were made with a rotary microtome (Leica RM2125). Then they were placed on glass slides, stained with periodic acid-Schiff’s reagent and mounted with synthetic Canada Balsam (Biopur, Buenos Aires, Argentina). Preparations were analysed using light microscope Leica DM5000B. Photographs were obtained using Leica DFC450, attached to the same microscope.

To prepare the samples for the observation by scanning electron microscopy (SEM), seed coat and endosperm were removed. The embryos were air-dried on filter paper for several seconds until the surface became dry (Barthlott, 1981). Then, backscattered electron (BSE) images were observed and micrographs of embryos were made using FEI Quanta 250 scanning electron microscope.

The embryogenesis stages were identified according to the embryogenesis of *Arabidopsis thaliana* (Park & Harada, 2008).

### Germination test

The germination of seeds of each variant was estimated by burying them in the soil at the experimental garden. Seeds collected earlier in the same year were germinated in identical boxes with peat. All the boxes were pierced for drainage, covered with agrofilm and buried at a depth of ∼10 cm. A total of 100 seeds of each variant (with three replications at least) were spread in each box. Seeds were buried at the beginning of October and left until April.

### Statistical analysis

The results submitted in the figures are represented as the mean of values ± standard error (SE). The effect of growth regulators treatment on germination of *H. sosnowskyi* seeds was tested in a one-way analysis of variance (Anova). For comparison of the means, a post hoc test (Tukey’s multiple range test) (*P* < 0.05) was used for significant differences.

## Results

### Effect of GA_3_ on formation and development of *Heracleumsosnowskyi* seeds

**Figure 2:**
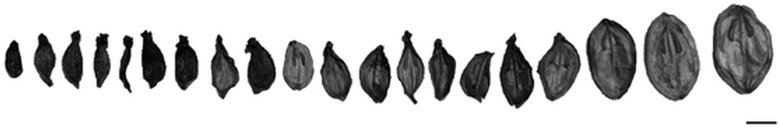
The variac line of GA_3_ treated *H. sosnowskyi* seeds. Scale bar, 5 mm.

To determine whether there were any morphological effects, *H. sosnowskyi* seeds treated with GA_3_ at concentration of 0.07 mM, 0.14 mM, 0.28 mM, 0.43 mM were compared with a control variant. It was noticed that gibberellin didn’t have significant effect on seed growth, as treatment with GA_3_ didn’t change their size. *H. sosnowskyi* inflorescences are compound umbrellas and the seed maturity depends on the seed area in the umbrella. Different time of maturity was the reason of different size of soaked and control variant seeds (Fig. 2).

**Figure 3:**
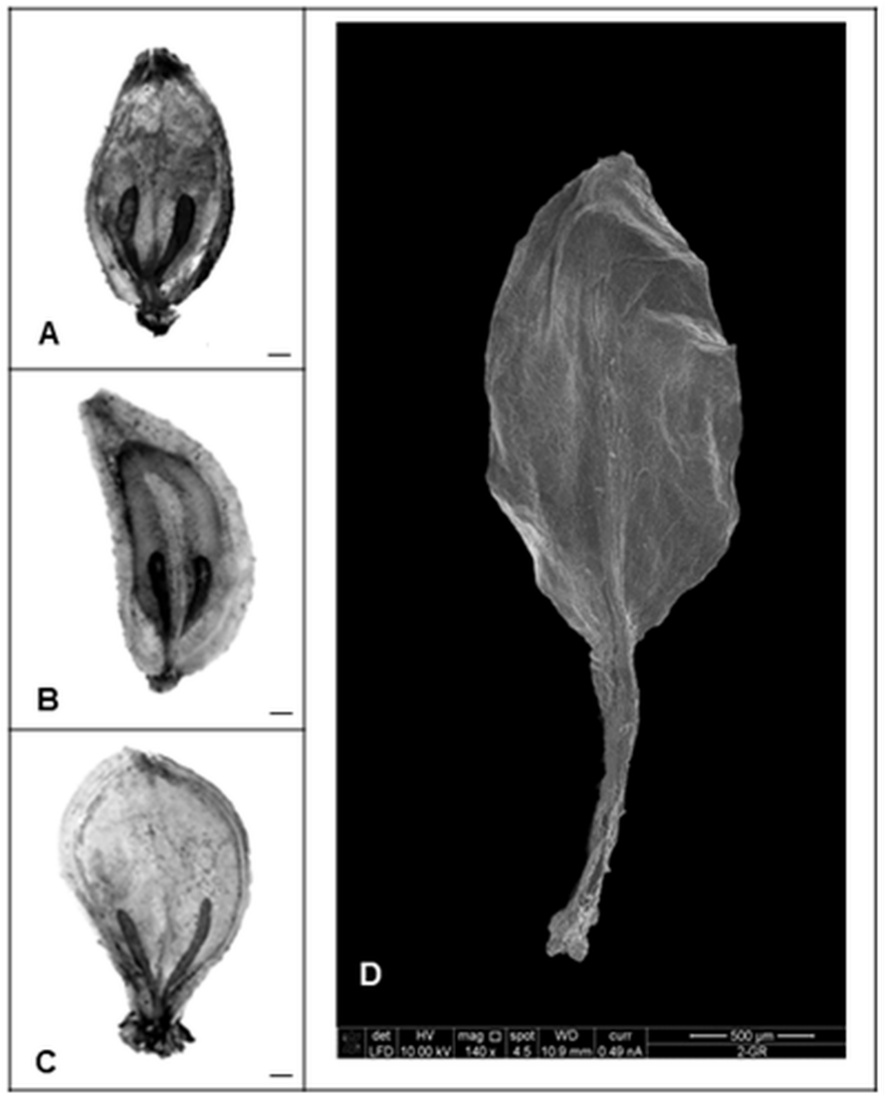
*Heracleum sosnowskyi* seed surfaces. (A) Without treatment (control). (B) GA_3_ (0.43 mM). (C) GA_3_ (0.14 mM). Scale bar, 1 mm. (D) Scanning micrograph of degenerated endosperm and embryo (the seed coat is removed). Scale bar, 500 µm.

Nevertheless, our studies indicated that the application of gibberellin induced different changes in morphology and histology of seeds. All control mature fruits of *H. sosnowskyi* were elliptic, laterally flattened, shallowly ridged schizocarps, composed of two one-seeded not-opening mericarps with swollen brown oil canals, which didn’t reach the base of both sides of mericarps (Fig. 3A).

The shape of most treated seeds changed (Figs. 3B and 3C). It was observed that in the case of higher GA_3_ concentration (0.43 mM), the seeds were not elliptic, but one side strongly convex (Fig. 3B). Lower concentrations didn’t cause such an effect. However, it was noticed that GA_3_ at concentration of 0.14 mM provoked widespread of stylopodium (Fig. 3C).

Beside all these remarks, it was determined that GA_3_ treatment in some seeds caused degeneration of endosperm and embryo (Fig. 3D). In spite of the fact that zygotic tissue didn’t form, the seed coat formed very well.

### Development of *H. sosnowskyi* seed embryo under GA_3_ treatment

**Figure 4:**
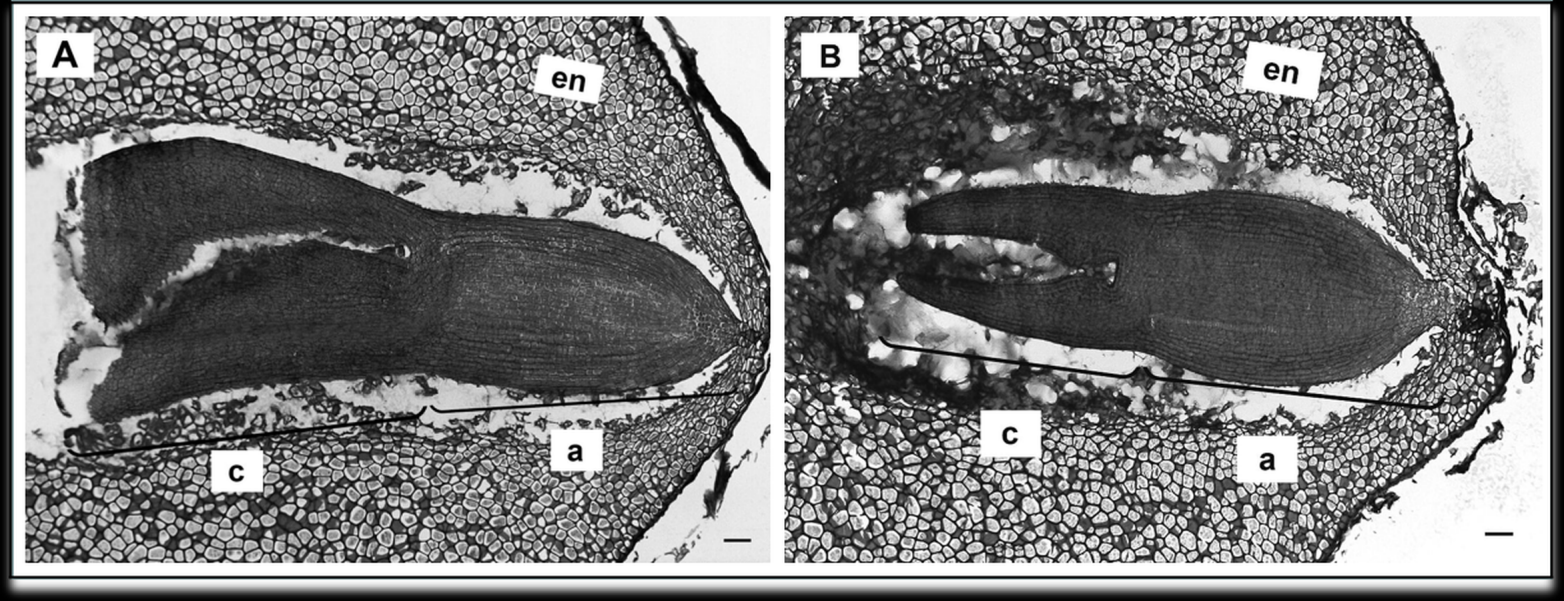
Light micrographs of longitudinal sections of *H. sosnowskyi* seed embryos. (A) Control. (B) After GA_3_ (0.43 Mm) application. Abbreviations: a, axis; c, cotyledons; en, endosperm. Scale bar, 50 µm.

To determine the effect of GA_3_ treatment on anatomical seed development, paraffin sections of GA_3_-treated and untreated seeds were analysed. Examination of longitudinal sections of control variant of *H. sosnowskyi* seeds showed that it contains large amount of endosperm, which surrounds mature embryo located at the micropylar end of the seed (Fig. 4A). It was noticed that exogenous GA_3_ treatment affected *Heracleum sosnowskyi* seed embryo development. A longitudinal section of seeds clearly revealed that embryo of seeds treated with GA_3_ at concentration of 0.43 mM was found to be at a torpedo stage (Fig. 4B).

However, in the seeds treated with GA_3_ at lower concentration, embryos at a torpedo stage were not observed.

**Figure 5:**
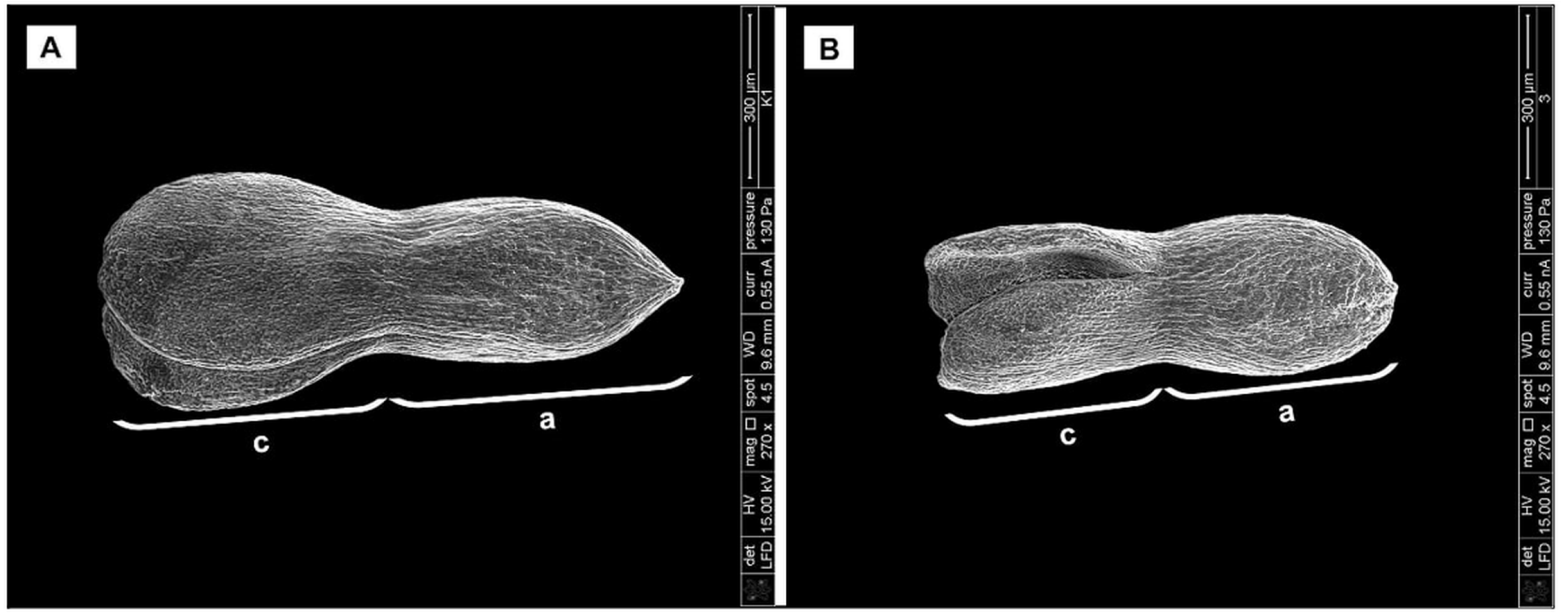
Scanning micrographs of embryos received from seed of *H. sosnowskyi*. Plants untreated (A) and treated with GA_3_ at a concentration of 0.43 mM (B). Abbreviations: a, axis; c, cotyledons. Scale bar, 300 µm.

Biometrical analysis of *H*. *sosnowskyi* embryos also showed that the embryos of control variant were significantly longer (Fig. 5A) than those treated with GA_3_ at concentration of 0.43 mM (Fig. 5B): 1070.35 ± 3.3 µm and 828.21 ± 3.4 µm, respectively (Student’s *t*-test, *p* < 0.05, values calculated on the basis of SEM micrographs).

Taken together, these results indicate that GA_3_ treatment can slowdown the development of embryo.

### Impact of different GA_3_ concentrations on the germination of *H. sosnowskyi* seeds

**Figure 6:**
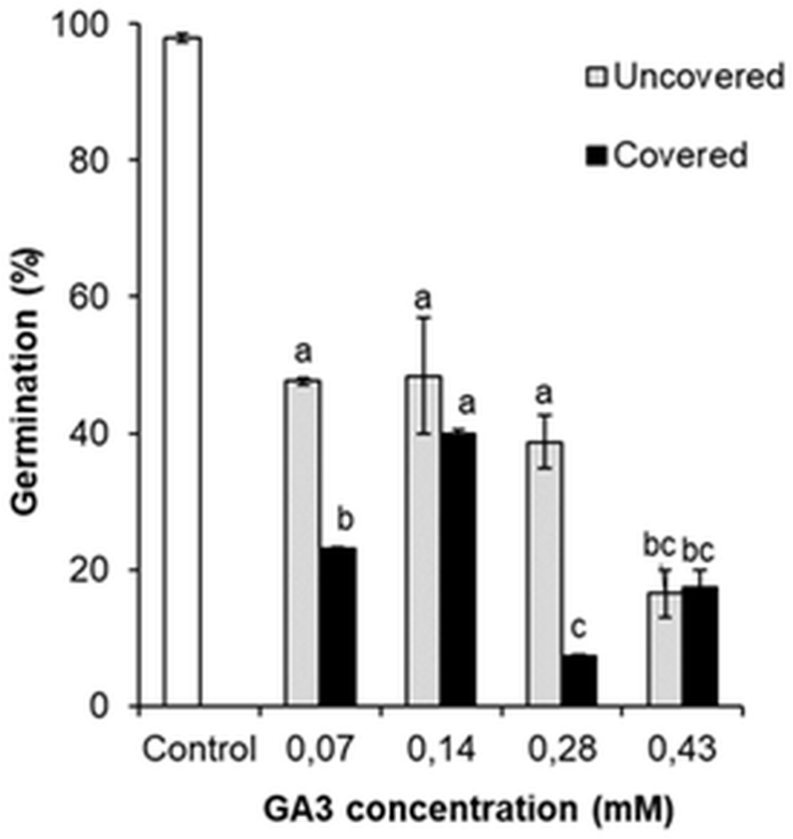
Germination of *H. sosnowskyi* seeds exposed to different concentrations (0.07 mM, 0.14 mM, 0.28 mM, 0.43 mM) of GA_3_ and covering with bags. Values indicate means ± SE. At least three replications. Different letters above histograms indicate significant difference at *P* < 0.05 (the Tukey HSD test).

The germination was analysed by comparing the percentage of GA_3_ treated (0.07 mM, 0.14 mM, 0.28 mM, 0.43 mM) and the control variant seeds. Comparison revealed that all GA_3_ concentrations reduced the germination of seeds (Fig. 6). It was observed that even low GA_3_ concentration (0.07 mM) significantly changed the percentage of seeds from 98.0% to 47.6%. However, the effect of GA_3_ (0.14 mM) treatment was almost the same (48.3%). The percentage of germinated seeds marginally reduced after GA_3_ (0.28 mM) application, but this change was not significant. The effect of GA_3_ (0.43 mM) was greater (16.5%) than that of GA_3_ (0.28 mM).

These results indicate that GA_3_ application reduces the germination of *H. sosnowskyi* seed, the effect depends on the concentration.

In addition, to check the impact of pollen-carrying insects on the germination of seeds after GA_3_ application, one part of the treated plants were covered with bags. It was determined that covering significantly changed the percentage of seed germination when GA_3_ concentrations of 0.07 mM, 0.28 mM were used. As compared to the not covered plants, the seed germination decreased by 24.6% and 31.5%, respectively (Fig. 6). However, it was determined that covering had no significant effect in the cases of GA_3_ 0.14 mM and GA_3_ 0.43 mM applications (Fig. 6).

### *H. sosnowskyi* seed germination under the effect of auxin transport inhibitor TIBA

**Figure 7:**
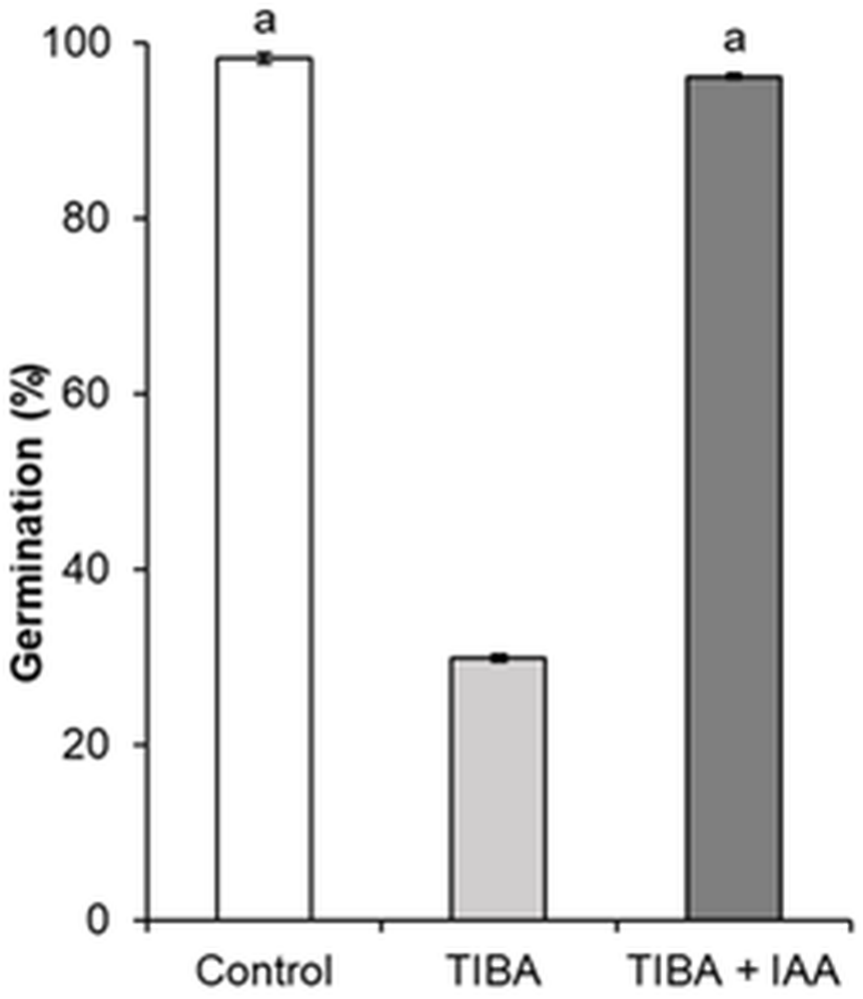
Germination of *H. sosnowskyi* seeds under the effect of auxin transport inhibitor TIBA. Values indicate means ± SE. At least three replications. The same letters above histograms indicate that means are not significantly different from each other. Histograms without letters indicate that means are significantly different from each other. Difference at *P* < 0.05 (the Tukey HSD test).

In order to show the influence of IAA and GA_3_ correlation on seed formation and germination, an inhibition of auxin transport by auxin transport inhibitor 2,3,5-triiodobenzoic acid (TIBA) was applied. As compared to control, the treatment with TIBA significantly changed the germination of seeds, from 98.1% to 29.8% (Fig. 7).

However, simultaneous application of auxin (IAA) and auxin transport inhibitor (TIBA) increased the seed germination and it almost reached the control variant (96.1%) (Fig. 7).

## Discussion

Phytohormones gibberellins (GA) are one group of plant growth regulators that play a prominent role in coordinating fruit growth and seed development. It is known that exogenous application of phytohormone GA makes changes in morphology and histology of fruit during its development or influences partenocarpic fruit formation (Serrani et al., 2007a; Serrani et al., 2007b; Serrani et al., 2010; Aparna & Remko, 2012; Fuentes et al., 2012; Cheng et al., 2013). Some studies have demonstrated that gibberellins influence the reduction of seed size (Cheng et al., 2013). Our results showed that exogenous application of GA_3_ had no significant effect on the size of *H. Sosnowskyi* seeds. Similar results have been obtained in the studies on fleshy fruit of *Capsicum annuum* L., where gibberellin had no additional effect on fruit growth. Treatment with GA_3_ alone does not change fruit size (Aparna & Remko, 2012).

Despite the fact that in our studies GA_3_ treatment did not change the size of *H. sosnowskyi* seeds, it was observed that the shape of all treated seeds varied one or another way. The effect depended only on GA_3_ concentrations (Figs. 3B and 3C). Our results showed that gibberellin GA_3_ application had effect on *H. sosnowskyi* seed embryo formation, too. It seems that such treatment (especially high levels of gibberellin concentration) slowed down the development of embryo (Figs. 4 and 5). Interestingly, it was remarked that in several treated mericarps, the seed coat formed very well, but they did not have endosperm and embryo (Fig. 3D). It is known that parthenocarpic fleshy fruit lack zygotic tissues. Many studies have demonstrated that incomplete seed development occurring as a result of embryo abortion and endosperm breakdown is caused by high levels of exogenous growth-promoting phytohormones near or at bloom (Iwahori, Weaver & Pool, 1968; Mesejo et al., 2008; Cheng et al., 2013). Based on such remarks, we put forward a hypothesis that *H. sosnowskyi* seeds without embryo and endosperm could be an example of parthenocarpy in the *Apiaceae* family fruits. Our results could supplement some studies, which show that the molecular mechanisms by which hormones regulate fruit set seems very similar in fleshy and dry fruit (Dorcey et al., 2009; Serrani et al., 2008).

The impacted seed germination is the indicator of treatment efficiency. Our results suggest the dependence of seed germination on the intensity of application. The highest GA_3_ concentration resulted in the lowest seed germination—from 98.0% (control) to 16.5% (GA_3_ concentration 0.43 mM) (Fig. 6). Such a result indicates that GA_3_ application may reduce *H. sosnowskyi* seed-bank as well as their seedling density. It is known that seedling density and population structure depends on the availability of viable seeds in the soil seed-bank (Moravcova et al., 2007a; Moravcova et al., 2007b; Gudžinskas & Žalneravičius, 2018).

Many studies have shown that fruit set of plants largely depends on the biosynthesis and crosstalk of phytohormones. It is known that there is a synergistic effect on growth in the case of combined action of GA and IAA (Serrani et al., 2008; Ding et al., 2013). To test the crosstalk between auxin and gibberellin, we studied the effect of TIBA on seed germination. A negative effect of TIBA on fruit growth and seed development has been observed by (Hamamoto et al., 1998; Serrani et al., 2010). In the present work, we used different procedures and our results indicated that auxin transport inhibitor TIBA reduced seed germination. Only joint treatment with IAA restored seed germination almost up to the control variant (Fig. 7).

It seems that by undermining auxin transport TIBA influences gibberellin synthesis, which results in negative germination of seeds.

In the present paper, we checked whether the covering of plants effects the germination of seeds. It was observed that there were significant differences between the covered and not covered variants, but the results were controversial (Fig. 6). As we expected, the covering of plants decreased seed germination. However, in one case (GA_3_ 0.14 mM), the influence of covering was observed, but difference was not statistically significant. Interestingly, it was remarked that under the effect of high concentration (0.43 mM) of GA_3_, the covering of plants did not change seed germination completely. Based on the obtained results, we think that GA_3_ concentration (0.43 mM) was quite high and made maximal effect on seed germination. Therefore, an additional impact was no longer relevant.

## Conclusions

Our results support the idea that external GA_3_ application inhibits *H. sosnowskyi* seed development and germination. This type of treatment could be used to stop the spread of this invasive plant because it certainly may reduce *H. sosnowskyi* seed-bank. So far, we cannot yet discuss parthenocarpy of the *Apiaceae* family plants because it needs more studies and could be a good field for our future research.

##  Supplemental Information

10.7717/peerj.6906/supp-1 File S1 Seed germination under the effect of exogenous application of TIBA and GA_3_

## Additional Information and Declarations

### Data Availability

The following information was supplied regarding data availability: The raw measurements are provided in File S1.
